# Machine learning approach using ^18^F-FDG-PET-radiomic features and the visibility of right ventricle ^18^F-FDG uptake for predicting clinical events in patients with cardiac sarcoidosis

**DOI:** 10.1007/s11604-024-01546-y

**Published:** 2024-03-16

**Authors:** Masatoyo Nakajo, Daisuke Hirahara, Megumi Jinguji, Satoko Ojima, Mitsuho Hirahara, Atsushi Tani, Koji Takumi, Kiyohisa Kamimura, Mitsuru Ohishi, Takashi Yoshiura

**Affiliations:** 1 Department of Radiology, Graduate School of Medical and Dental Sciences, Kagoshima University, 8-35-1 Sakuragaoka, Kagoshima, 890-8544 Japan; 2 Department of Management Planning Division, Harada Academy, 2-54-4 Higashitaniyama, Kagoshima, 890-0113 Japan; 3 Department of Cardiovascular Medicine and Hypertension, Graduate School of Medical and Dental Sciences, Kagoshima University, 8-35-1 Sakuragaoka, Kagoshima, 890-8544 Japan

**Keywords:** Cardiac sarcoidosis, ^18^F-FDG, PET/CT, Machine learning, Adverse clinical events

## Abstract

**Objectives:**

To investigate the usefulness of machine learning (ML) models using pretreatment ^18^F-FDG-PET-based radiomic features for predicting adverse clinical events (ACEs) in patients with cardiac sarcoidosis (CS).

**Materials and methods:**

This retrospective study included 47 patients with CS who underwent ^18^F-FDG-PET/CT scan before treatment. The lesions were assigned to the training (*n* = 38) and testing (*n* = 9) cohorts. In total, 49 ^18^F-FDG-PET-based radiomic features and the visibility of right ventricle ^18^F-FDG uptake were used to predict ACEs using seven different ML algorithms (namely, decision tree, random forest [RF], neural network, k-nearest neighbors, Naïve Bayes, logistic regression, and support vector machine [SVM]) with tenfold cross-validation and the synthetic minority over-sampling technique. The ML models were constructed using the top four features ranked by the decrease in Gini impurity. The AUCs and accuracies were used to compare predictive performances.

**Results:**

Patients who developed ACEs presented with a significantly higher surface area and gray level run length matrix run length non-uniformity (GLRLM_RLNU), and lower neighborhood gray-tone difference matrix_coarseness and sphericity than those without ACEs (each, *p* < 0.05). In the training cohort, all seven ML algorithms had a good classification performance with AUC values of > 0.80 (range: 0.841–0.944). In the testing cohort, the RF algorithm had the highest AUC and accuracy (88.9% [8/9]) with a similar classification performance between training and testing cohorts (AUC: 0.945 vs 0.889). GLRLM_RLNU was the most important feature of the modeling process of this RF algorithm.

**Conclusion:**

ML analyses using ^18^F-FDG-PET-based radiomic features may be useful for predicting ACEs in patients with CS.

**Supplementary Information:**

The online version contains supplementary material available at 10.1007/s11604-024-01546-y.

## Introduction

Sarcoidosis is a systemic granulomatous inflammatory disease of unknown etiology. Cardiac involvement is clinically rare, and it only occurs in 5% of patients with sarcoidosis [1, 2]. However, cardiac sarcoidosis (CS) is an important predictor of poor prognosis in patients with sarcoidosis due to complications such as atrioventricular block (AVB), ventricular tachycardia (VT), and congestive heart failure [3–5]. Thus, it is extremely important to make early diagnosis and evaluate disease activity for managing patients with CS [6, 7].

Glucose metabolic activity can be evaluated by measuring ^18^F-FDG uptake during PET/computed tomography (CT) scan for not only oncological but also inflammatory disorders [8, 9]. However, only a few studies have used ^18^F-FDG-PET-based radiomic features for diagnosing or predicting treatment response in CS [10, 11]. Recently, the potential applications of machine learning (ML) analysis have been reported in the field of nuclear cardiology [12–14]. However, to the best of our knowledge, no study has examined the efficacy of the ML approach using ^18^F-FDG-PET-/CT-based radiomics on predicting adverse clinical events (ACEs) in patients with CS.

The current study aimed to investigate the usefulness of ML models using pretreatment ^18^F-FDG-PET-based radiomic features for predicting the risk of ACEs in patients with CS.

## Materials and methods

### Patients

This retrospective study was approved by the institutional review board, and the need for informed consent was waived. In total, 70 consecutive patients with known or suspected CS underwent pretreatment ^18^F-FDG-PET/CT scan from April 2012 to December 2022. Their clinical records were reviewed to identify patients who should be evaluated.

In a previous study [15], the usefulness of Patlak Ki images extracted from dynamic ^18^F-FDG-PET/CT scan for evaluating the risk of clinical events in CS was examined. The previous study enrolled 21 patients with CS who underwent 30 ^18^F-FDG-PET/CT scan, which included pretreatment, undertreatment, and follow-up scans, between April 2019 and January 2020. However, analyses using ML approaches for predicting the risk of ACEs in patients with CS using pretreatment ^18^F-FDG-PET-based radiomic features were not performed. Thus, among 21 patients, 8 with pretreatment ^18^F-FDG-PET/CT scans were included in the current study. The inclusion criteria were as follows: (1) patients diagnosed with CS according to the Japanese Society of Sarcoidosis and Other Granulomatous Disorders guidelines [16], (2) those without a history of steroid treatment, and (3) those with visible cardiac ^18^F-FDG uptake on PET/CT scan. The exclusion criteria were patients with a history or coexistence of other cardiac disorders.

Of 70 patients, 12 without cardiac ^18^F-FDG uptake were excluded. Among the remaining 58 patients, 11 were further excluded because of hypertrophic cardiomyopathy (*n* = 2), dilated cardiomyopathy (*n* = 2), ventricular aneurysm (*n* = 1), and lack of CS evidence (*n* = 6).

Finally, 47 patients (38 women and 9 men; mean age: 61 ± 10 [age: 39–81] years) were eligible for the analyses. Immunosuppressive treatment was adopted for these patients after the pretreatment ^18^F-FDG-PET/CT scan according to the recommendations of the Japanese Society of Sarcoidosis and Other Granulomatous Disorders guidelines [16]. The loading dose of prednisolone was 30 mg/day, which was tapered to a maintenance dose and administrated to all patients during the follow-up period.

### Imaging protocols

All patients were instructed to follow a high-fat and low-carbohydrate diet for 1 day, and followed by a fast of at least 18 h before ^18^F-FDG-PET/CT scan, which resulted in a mean plasma glucose level of 102 (range: 71–154) mg/dL immediately before intravenous ^18^F-FDG administration.

All ^18^F-FDG-PET/CT scan procedures were performed using two whole-body PET/CT scanners. The Discovery 600M PET/CT scanner (GE Healthcare, Milwaukee, WI, the USA) was used from April 2012 to January 2018 and the Discovery MI scanner (GE Healthcare) from February 2018 to December 2022. The emission scan was performed 1 h after the administration of ^18^F-FDG (mean: 223 ± 30 [155–277] MBq) after CT data acquisition (slice thickness: 3.75 mm, pitch: 1.375 mm, 120 keV, auto mA: 40–100 mA, based on body mass, and reconstructed matrix size: 512 × 512). The acquisition time was 2.5 min per bed position (total: 7–11). Attenuation-corrected data were acquired. Using the Discovery 600M scanner, images were reconstructed with a three-dimensional ordered subset expectation–maximization algorithm (image matrix size: 192 × 192, 16 subsets, two iterations, voxel size: 3.125 × 3.125 × 3.27 mm^3^, and VUE Point Plus). Using the Discovery MI scanner, a Bayesian penalized likelihood reconstruction algorithm was used (image matrix size: 192 × 192, voxel size: 2.60 × 2.60 × 2.78 mm^3^, penalization factor: 700, and Q. Clear) with the point spread function. Each scanner used a consistent reconstruction setting and matrix.

### Image and radiomic feature analyses

Two radiologists (with 12 and 20 years of ^18^F-FDG-PET/CT scan experience) who were knowledgeable about the study purpose but were blinded to the clinical information read the ^18^F-FDG PET/CT scan images. The radiologists visually assessed each ^18^F-FDG-PET/CT scan image as negative (myocardial visibility lower than or similar to that of the liver) or positive (myocardial visibility higher than that of the liver) ^18^F-FDG uptake [17] in the left ventricle (LV) and right ventricle (RV) myocardium. In case of a disagreement, they reached a consensus.

A third radiologist (18 years of ^18^F-FDG-PET/CT experience) performed quantitative analyses of the visible myocardial lesions. The third radiologist generated the volume of interest (VOI) by manually placing a region of interest on a suitable reference-fused axial image, and defined the craniocaudal and mediolateral extents encompassing the whole positive myocardial lesion, excluding any avid extracardiac structures. Next, the maximum standardized uptake value (SUV_max_) threshold was set at 40%, which was commonly used in previous studies [18], to automatically delineate a VOI equal to or greater than the 40% threshold of SUVmax. The LIFEx package (version 6.00) [19] was used to extract 49 radiomic features from PET images (Supplemental Table 1). The LIFEx package is used to calculate textural features only for VOIs of at least 64 voxels. These 49 radiomic features were included in five categories (shape and first-order characteristics, gray level co-occurrence matrix, neighborhood gray-tone difference matrix [NGTDM], gray level run length matrix [GLRLM], and gray level zone length matrix). The VOI and SUV were resampled into discrete bins using absolute resampling to minimize the correlation between textural features and reduce the impact of noise and matrix size [20]. Sixty-four bins were used for the PET component with the minimum and maximum bounds of the resampling interval set to SUVs of 0 and 20, respectively. Moreover, the voxel size was resampled to 3.0 × 3.0 × 3.0 mm^3^. Therefore, a bin size with an SUV of 0.3 was used to analyze the PET component. Voxels with an SUV of > 20 were grouped in the highest bin [20].

As we used two different PET scanners, post-reconstruction harmonization was performed for all PET parameters using the ComBat harmonization method for R software (https://github.com/Jfortin1/ComBatHarmonization) [21], which is effective in PET scans [22].

### Confirmation of ACEs

Echocardiography was performed within 2 months of ^18^F-FDG-PET/CT scan (mean ± standard deviation: 13 days ± 14 [range: − 50 to + 58 days]). The echocardiography report was used as the reference standard for cardiac function. Cardiac dysfunction was defined as a LV ejection fraction (LVEF) of < 50% [23]. Further, twelve-lead or Holter echocardiography was performed within 2 months of ^18^F-FDG-PET/CT scan (mean ± standard deviation: 17 days ± 15 [range: − 50 to + 58 days]). Moreover, patients were assessed to determine the presence of arrhythmic events, including sustained VT and AVB. AVB was characterized as either second- or third-degree AVB or trifascicular block [23, 24].

Medical records were used to obtain information on patient prognosis. The last follow-up was conducted in December 2023. ACE was defined as the reduction in LVEF with cardiac dysfunction (LVEF of < 50%), hospitalization due to cardiac arrhythmia such as recurrence or onset of sustained VT and AVB or heart failure, and death [25, 26]. Change in LVEF was determined by comparing the findings between echocardiography studies performed nearest to the pretreatment PET study and the last echocardiography studies of the follow-up period. Decrease in LVEF was defined as a negative change in LVEF.

### ML approach

We adopted 49 radiomic features and the visibility of RV ^18^F-FDG uptake to predict ACEs using the ML approaches. Data were stratified according to event and were randomly assigned into the training (80%) and testing (20%) cohorts. Based on the ML analysis for predicting ACEs, decision tree, random forest (RF), neural network, k-nearest neighbors (kNN), Naïve Bayes, logistic regression (LR), and support vector machine (SVM), which are popular ML algorithms, were used for binary classification [27, 28].

The parameter selection for each ML method in this study was carefully made based on the specific clinical challenges and the characteristics of our dataset. For the decision tree, we limited node levels and split thresholds to prevent overfitting, and consequently we selected an induce binary tree with two minimum number of instances in leaves, a split greater than 5, with maximum 100 node levels for depth of classification tree and stop splitting the nodes after majority reach 95%. In the RF, a moderate number of trees were chosen to balance the model’s generalizability and computational efficiency, and consequently we selected 10 trees and did not split subsets smaller than 5. The neural network settings were optimized with rectified linear unit (ReLU) activation function and Adam optimization for efficient learning and good convergence, and consequently we selected 1000 neurons, alpha = 0.00001 and maximum iterations 1000. For kNN, setting the number of neighbors to 5 with metric Euclidean and weight uniform ensured suitable accuracy for our dataset size. The parameters for LR and SVM were chosen to optimize the tradeoff between model complexity and the risk of overfitting. Consequently, we selected a ridge with a coefficient score of 1 for LR. For SVM, we selected the Kernel radial basis function with cost 1 and regression loss epsilon 0.10, and the two optimization parameters, tolerance and iteration limit were set to 0.0010 and 500, respectively. In the case of Naïve Bayes, its simplicity and effective learning ability based on the distribution of data were valued. These parameter choices enabled us to construct robust and reliable predictive models aligned with the objectives of our study.

To overcome imbalanced data, the synthetic minority over-sampling technique was used in the training cohorts [29]. In this study, the sample size was small, and the set of features was reduced to prevent the influence of overfitting. The ranking-based method was only applied on the training cohort to reduce set features based on the decrease in Gini impurity. As a rule of thumb, it is necessary to use < 10% of the sample size as the number of features for classification problem [30]. The final sample size of this study was *n* = 47; thus, we selected the 4 top ranking features for constructing each ML model. Moreover, the use of a resampling technique referred to as *k*-fold cross-validation is one of the solutions of overfitting [31, 32]. Tenfolds are a common choice for *k*-fold cross-validation, particularly if the dataset is not extremely large or small [32]. In this study, a tenfold cross-validation was used to minimize the negative influence of overfitting on the training cohort.

Receiver operating characteristic curve (ROC) analysis was performed to compare the predictive performances of the models, and the area under the ROC curve (AUC) was calculated. The computed performance measures were AUC, accuracy, *F*1 score, precision (positive predictive value), and recall (sensitivity) for average over classes. The *F*1 score (*F* score or *F* measure) is the harmonic average between precision and recall [33]. Each ML algorithm was used to calculate each probability score (range: 0–1) of ACEs. The predictive performance of each machine model was independently estimated in the testing set by quantifying the AUC, accuracy, *F*1 score, precision, and recall.

The diagnostic indices including sensitivity, specificity, positive predictive value (PPV), and negative predictive value (NPV) of the testing cohort were also calculated. The importance of features in the ML modeling process was calculated using the decrease in AUC [34]. A higher decrease in AUC for a feature indicates that such a variable has a higher importance [34].

The ML analysis was performed using Orange version 3.24.1 (Bioinformatics Laboratory, University of Ljubljana, Ljubljana, Slovenia), an open-source data-mining and visualization package [35].

### Statistical analysis

The Mann–Whitney *U* test or the Chi-square test was used to appropriately assess differences between two quantitative variables or compare categorical data. The DeLong method was used to analyze the statistical significance of differences between AUCs [36]. The diagnostic indices including sensitivity, specificity, PPV, NPV, and accuracy were compared using the McNemar’s test or Chi-square test.

Data were presented as medians and interquartile ranges (IQRs). A *p* value of < 0.05 was considered statistically significant, and all *p* values were two-tailed. The MedCalc statistical software (MedCalc Software Ltd., Acacialaan 22, 8400 Ostend, Belgium) was used for statistical analyses.

## Results

### Characteristics of the patients

Of 47 patients, the median LVEF was 50.0% (IQR: 38.3%–63.8% [range: 20.8–81.0%]), and cardiac dysfunction was observed in 22 patients and arrhythmic events in 16 patients before treatment. There were seven patients with positive RV ^18^F-FDG uptake on the pretreatment ^18^F-FDG-PET/CT scans. The mean follow-up duration was 48.6 (range: 7–139) months. Of 47 patients, 17 presented with ACEs during follow-up: 11 patients were hospitalized because of cardiac arrhythmia (*n* = 6) and heart failure (*n* = 5), five patients had worsening of systolic LV function and one patient died. The complication rate of ACEs was significantly higher in patients with positive RV ^18^F-FDG uptake than in patients with negative RV ^18^F-FDG uptake (85.7% [6/7] vs. 27.5% [11/40], *p* = 0.006).

Table 1 shows the clinical characteristics of the participants in the training and testing cohorts. Of 38 patients in the training cohort, the median LVEF was 49.0% (IQR: 36.5–63.9% [range: 20.8–81.0%]) and cardiac dysfunction was observed in 19 patients and arrhythmic events in 13 patients before treatment. There were seven patients with positive RV ^18^F-FDG uptake on the pretreatment ^18^F-FDG-PET/CT scans. Fourteen patients developed ACEs during follow-up: eight patients were hospitalized because of cardiac arrhythmia (*n* = 4) or heart failure (*n* = 4), five patients had worsening of systolic LV function, and one patient died.

**Table 1:** Characteristics of patients with cardiac sarcoidosis (*n* = 47)

Characteristics	Training cohort (*n* = 38)			Testing cohort (*n* = 9)			*p* Value^a^
	Median	IQR	Range	Median	IQR	Range	
LVEF (%)	49.0	36.5–63.9	20.8–81.0	54.4	43.9–64.0	30.3–74.1	0.51
	Number			Number			
Cardiac function							
Dysfunction (LVEF < 50%)		19			3		0.37
Normal (LVEF > 50%)		19			6		
Arrhythmic events							
Presence		13			3		0.96
Absence		25			6		
RV ^18^F-FDG uptake							
Positive		7			0		0.17
Negative		31			9		
Adverse clinical events							
Presence		14			3		0.85
Absence		24			6		

*IQR*, interquartile range; *LVEF*, left ventricular ejection fraction; *RV*, right ventricle

^a^Comparison of the training and testing cohorts

Of nine patients in the testing cohort, the median LVEF was 54.4% (IQR: 43.9–64.0% [range: 30.3–74.1%]), and cardiac dysfunction was observed in three patients and arrhythmic events in three patients before treatment. Three patients developed ACEs during follow-up: all three patients were hospitalized because of cardiac arrhythmia (*n* = 2) or heart failure (*n* = 1).

No significant differences were observed in terms of LVEF, cardiac dysfunction, arrhythmic events, RV ^18^F-FDG uptake, and ACEs between the training and testing cohorts (each, *p* > 0.05).

### ML models for predicting ACEs

Radiomic features were ranked based on the decrease in Gini impurity (Supplemental Table 2). The top four features for predicting ACEs were surface area, GLRLM_RLNU, coarseness from the NGTDM (NGTDM_Coarseness), and sphericity. Patients who experienced ACEs had a significantly higher surface area (*p* < 0.001), GLRLM_RLNU (*p* < 0.001) and a lower NGTDM_Coarseness (*p* = 0.002) and sphericity (*p* = 0.010) than those without ACEs (Table 2).

**Table 2:** Comparison of the top four radiomic predictive features between patients with cardiac sarcoidosis who developed adverse clinical events and those who did not

Features	Adverse clinical events						*p* Value
	Patients without adverse clinical events (*n* = 30)			Patients with adverse clinical events (*n* = 17)			
	Median	IQR	Range	Median	IQR	Range	
Surface area (mm^2^)	19,458.2	9426.3–22,243.3	851.1–45,417.8	38,427.9	25,277.1–41,865.1	12,681.6–58,135.6	< 0.001
GLRLM_RLNU	1780.3	1129.4–2402.3	131.8–4813.5	3707.6	2913.5–4542.9	1170.5–6338.9	< 0.001
NGTDM Coarseness (× 10^−3^)	2.4	1.7–4.5	0.9–30.2	1.4	0.7–2.0	0.2–23.8	0.002
Sphericity	0.55	0.49–0.68	0.39–0.85	0.47	0.40–0.57	0.28–0.78	0.010

*IQR*, interquartile range; *GLRLM*, gray level run length matrix; *RLNU*, run length non-uniformity; *NGTDM*, neighborhood gray-tone difference matrix

The ML model was constructed using these top four features to prevent overfitting. Table 3 presents the diagnostic performance of each ML algorithm in the training and testing cohorts to predict ACEs.

**Table 3:** Diagnostic performance of each machine learning model using the top four radiomic features for predicting adverse clinical events in the training and testing cohorts

Algorithm	Training cohort (average over classes)					Testing cohort								
						Average over classes				Predicting for adverse clinical events				
	AUC	F1	Precision	Recall	Accuracy	AUC	F1	Precision	Recall	Sensitivity	Specificity	PPV	NPV	Accuracy
Decision tree	0.841	0.812	0.817	0.813	0.813	0.750	0.778	0.778	0.778	66.7% (2/3)	83.3% (5/6)	66.7% (2/3)	83.3% (5/6)	77.8% (7/9)
RF	0.935	0.875	0.878	0.875	0.875	0.889	0.882	0.905	0.899	66.7% (2/3)	100% (6/6)	100% (2/2)	85.7% (6/7)	88.9% (8/9)
Neural network	0.944	0.874	0.886	0.875	0.875	0.889	0.778	0.778	0.778	66.7% (2/3)	83.3% (5/6)	66.7% (2/3)	83.3% (5/6)	77.8% (7/9)
kNN	0.842	0.792	0.792	0.792	0.792	0.750	0.778	0.778	0.778	66.7% (2/3)	83.3% (5/6)	66.7% (2/3)	83.3% (5/6)	77.8% (7/9)
Naïve Bayes	0.907	0.766	0.796	0.771	0.771	0.778	0.778	0.778	0.778	66.7% (2/3)	83.3% (5/6)	66.7% (2/3)	83.3% (5/6)	77.8% (7/9)
LR	0.889	0.854	0.854	0.854	0.854	0.667	0.778	0.778	0.778	66.7% (2/3)	83.3% (5/6)	66.7% (2/3)	83.3% (5/6)	77.8% (7/9)
SVM	0.863	0.812	0.817	0.813	0.813	0.722	0.778	0.778	0.778	66.7% (2/3)	83.3% (5/6)	66.7% (2/3)	83.3% (5/6)	77.8% (7/9)

*RF,* random forest; *kNN* k-nearest neighbors; *LR,* logistic regression; *SVM*, support vector machine; *AUC*, area under the receiving operating characteristic curve; *PPV* positive predictive value; *NPV* negative predictive value

In the training cohort, all ML algorithms achieved AUC values of > 0.80 for predicting ACEs (range: 0.841–0.944). Moreover, 5 of 7 ML algorithms (decision tree, RF, neural network, LR, and SVM) achieved F1 scores (range: 0.812–0.875), precision (range: 0.817–0.886), recall (range: 0.813–0.875), and accuracy (range: 0.813–0.875) of > 0.80 for predicting ACEs.

In the testing cohort, RF and neural network algorithms had an AUC of > 0.80 for predicting ACEs. The classification performance of RF (AUC—training cohort: 0.935, testing cohort: 0.889) and neural network (AUC—training cohort: 0.944, testing cohort: 0.889) in the testing cohort was similar to that of the training cohort. Meanwhile, the performance of the remaining five ML algorithms was poorer in the testing cohort (AUCs: 0.667–0.778) than in the training cohort.

The diagnostic indices including sensitivity, specificity, PPV, NPV, accuracy, and AUC did not significantly differ among these seven ML algorithms (each, *p* > 0.05) (Supplemental Table 3). However, among the seven ML algorithms, RF had the highest diagnostic index (average over classes—AUC: 0.889, *F*1 score: 0.882, precision: 0.905, recall: 0.899, sensitivity: 66.7% [2/3], specificity: 100% [6/6], PPV: 100% [2/2], NPV: 85.7% [6/7], and accuracy: 88.9% [8/9]). Supplemental Fig. 1 shows the important features of RF calculated using the decrease in AUC. GLRLM_RLNU was the most important feature with the highest mean value (0.150) and had a higher contribution in the modeling process.

**Fig. 1:**
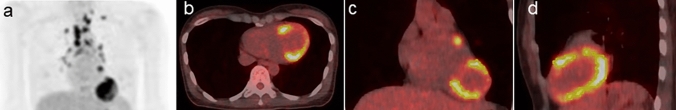
A 39-year-old female patient with cardiac sarcoidosis who developed ACEs (VT) after the immunosuppressive treatment with prednisolone. Pretreatment ^18^F-FDG-PET/CT scan [maximum intensity projection [MIP] (**a**), trans-axial (**b**), coronal (**c**), and sagittal (**d**)] images revealed ^18^F-FDG uptake in the sarcoidosis lesions of the lymph nodes (supra clavicular, hilar, and mediastinal region). The yellow line represents the border of the volume of interest in the myocardium (SUVmax 11.3 g/mL, SUVmean 6.1 g/mL, CMV 29.7 mL, and CMA 180.9 g). Thereafter, the immunosuppressive treatment with prednisolone was initiated. The ACE (VT) occurred 25 months after pretreatment ^18^F-FDG-PET/CT scan at a maintenance prednisolone dose of 10 mg/day. The calculated probability score for predicting the risk of ACEs (positive ≥ 0.5) was 0.90 on RF. Thus, the ML model with RF algorithm can predict the risk of ACEs in this case

**Fig. 2:**
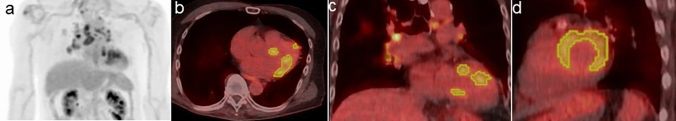
An 81-year-old male patient with cardiac sarcoidosis who did not develop ACEs after the immunosuppressive treatment with prednisolone. Pretreatment ^18^F-FDG-PET/CT scan [maximum intensity projection (MIP) (**a**), trans-axial (**b**), coronal (**c**), and sagittal (**d**)] images revealed ^18^F-FDG uptake in the sarcoidosis lesions of lymph nodes (hilar, and mediastinal region) and myocardium. The yellow line represents the border of the volume of interest in the myocardium (SUVmax 5.7 g/mL, SUVmean 4.0 g/mL, CMV 28.6 mL, and CMA 115.1 g). Thereafter, the immunosuppressive treatment with prednisolone was initiated. The ACE did not occur 30 months after pretreatment ^18^F-FDG-PET/CT scan at a maintenance prednisolone dose of 10 mg/day. The calculated probability score for predicting the risk of ACEs (positive ≥ 0.5) was 0 on RF. Thus, the ML model with the RF algorithm can predict the absence of ACE risk in this case

Figures 1 and 2 show the representative ^18^F-FDG-PET/CT images of patients with and without ACEs, respectively.

## Discussion

The current study evaluated the usefulness of the ML approach using pretreatment ^18^F-FDG-PET-based radiomic features and the visibility of RV ^18^F-FDG uptake for predicting ACEs in patients with CS. RF had the best performance for predicting ACEs, with the highest AUC and accuracy among all ML algorithms. GLRLM_RLNU had the highest contribution in the modeling process of RF. Therefore, ML analyses using ^18^F-FDG-PET-based radiomic features may be useful for predicting the risk of ACEs in patients with CS.

Previous studies have examined the characteristics of ^18^F-FDG-PET/CT radiomic features in CS. Manabe et al. [10] evaluated the diagnostic value of ^18^F-FDG-PET/CT texture analysis in patients with CS. Results showed that GLRLM long-run emphasis and GLRLM short-run low gray level emphasis were significant independent predictors of CS diagnosis. Moreover, their group examined the efficacy of ^18^F-FDG-PET/CT texture analysis on providing prognostic information on patients with CS. Moreover, they reported that GLRLM high gray level run emphasis was significantly associated with ACEs [11].

In our study, patients with CS who developed ACEs had a significantly higher surface area, GLRLM_RLNU, and a lower NGTDM_Coarseness and sphericity than those who did not. GLRLM_RLNU is one of the higher order texture features, and it measures differences between the lengths of runs. The high GLRLM_RLNU values are indicative of heterogeneous images [37]. Coarseness, which is one of the NGTDMs, is associated with granularity within an image and is related to the level of special rate of change in intensity. The heterogeneous images had a high rate of change in the gray level within a neighborhood, which results in a low coarseness value [38, 39]. Surface area represents the area of the surface encompassing the VOI and has a direct relationship with spiculatedness [40]. The sphericity represents the degree to which the VOI is similar to a sphere (formula of calculation of sphericity was presented in the Supplemental Material), and sphericity increases as the shape of VOI more closely resembles that of a sphere [41]. Thus, ACEs may occur in patients with CS as evidenced by a more heterogeneous and larger myocardial ^18^F-FDG uptake, and higher asphericity.

Recently, the potential applications of ML analysis have been reported in the field of nuclear cardiology [12–14]. Hu et al. [12] examined the usefulness of ML models for predicting early coronary revascularization after single-photon emission computed tomography (SPECT) myocardial perfusion imaging (MPI). Results showed that the ML model outperformed the expert interpretation of MPI by nuclear cardiologists for predicting early revascularization performance. Rios et al. [13] showed that the ML models using automatically extracted variables had a better prognostic accuracy for major cardiac ACEs compared with standard interpretation in patients undergoing SPECT MPI. However, to the best of our knowledge, no study has previously investigated the efficacy of ^18^F-FDG-PET-based radiomics and the visibility of RV ^18^F-FDG uptake using the ML approach for predicting ACEs in patients with CS.

In our study, to prevent the influence of overfitting, the ML models were constructed using the top four features ranked by the decrease in Gini impurity to predict ACEs. In the training cohort, all seven ML algorithms had a good classification performance with AUC values of > 0.80. However, in the testing cohort, only two algorithms with RF and neural network algorithm achieved AUC values of > 0.80. Meanwhile, the performance of the remaining five ML algorithms (decision tree, kNN, Naïve Bayes, LR, and SVM) was poorer in the testing cohort (AUCs of 0.667–0.778) than in the training cohort probably due to overfitting. Although neither the AUC nor accuracy significantly differed among the seven ML algorithms, RF was the best performing classifier as it had the highest diagnostic accuracy (88.9% [8/9]). Moreover, it exhibited a similar classification performance between the training and testing cohorts (AUC: 0.935 vs 0.889). GLRLM_RLNU was the most important feature for the ML modeling process of RF. Hence, the ML model with RF algorithm using ^18^F-FDG-PET-based radiomic features and the visibility of RV ^18^F-FDG uptake can potentially predict ACEs in patients with CS.

It has been reported that ^18^F-FDG accumulation in the RV is associated with the ACEs [25, 42]. In our study, the complication rate of ACEs was significantly higher in patients with positive RV ^18^F-FDG uptake than that of patients with negative RV ^18^F-FDG uptake (85.7% [6/7] vs. 27.5% [11/40], *p* = 0.006). Thus, this finding was compatible with the previous reports [25, 42]. However, the visibility of RV ^18^F-FDG uptake was not ranked within top four features, and the constructed each ML model was not influenced by the visibility of RV ^18^F-FDG uptake.

This study had several limitations. First, it was retrospective in nature, and it had a relatively small study cohort with conducting only in a single institution. Thus, it is necessary to perform a multicenter prospective study with a significantly larger population to validate and confirm our findings. Second, using different PET/CT scanners might have affected the results of ^18^F-FDG-PET-based radiomic analyses. However, the post-reconstruction harmonization using ComBat was conducted during analyses to overcome this issue. Third, only 49 radiomic features extracted from the LIFEx software were used in ML analyses. However, the LIFEx software has been widely used for radiomic analyses in the field of PET/CT scan studies [43, 44]. Fourth, only seven ML algorithms (specifically decision tree, RF, neural network, kNN, Naïve Bayes, logistic regression, and SVM) were applied in the ML analyses. Nevertheless, we only used the ML algorithms implemented in the Orange software, which is a popular open-source tool that provides a visual approach to ML for an interactive data analysis, thereby facilitating the easy construction and configuration of workflows for ML studies [35]. Finally, although training and testing validation had a good classification performance, a training–test scheme with a larger population might be preferred for model validation.

In conclusion, ML analyses using ^18^F-FDG-PET-based radiomic features can be useful for predicting ACEs in patients with CS.

### Supplementary Information

Below is the link to the electronic supplementary material.

Supplementary file1 (DOCX 116 KB)

## References

[1] Hulten E, Aslam S, Osborne M (2016). Cardiac sarcoidosis: state of the art review. Cardiovasc Diagn Ther.

[2] Doughan AR, Williams BR (2006). Cardiac sarcoidosis. Heart.

[3] Banba K, Kusano KF, Nakamura K (2007). Relationship between arrhythmogenesis and disease activity in cardiac sarcoidosis. Heart Rhythm.

[4] Roberts WC, McAllister Jr HA, Ferrans VJ (1977). Sarcoidosis of the heart. A clinicopathologic study of 35 necropsy patients (group 1) and review of 78 previously described necropsy patients (group 11). Am J Med.

[5] Yazaki Y, Isobe M, Hiroe M (2001). Prognostic determinants of long-term survival in Japanese patients with cardiac sarcoidosis treated with prednisone. Am J Cardiol.

[6] Mehta D, Lubitz SA, Frankel Z (2008). Cardiac involvement in patients with sarcoidosis: diagnostic and prognostic value of outpatient testing. Chest.

[7] Ishida Y, Yoshinaga K, Miyagawa M (2014). Recommendations for (18)F-fluorodeoxyglucose positron emission tomography imaging for cardiac sarcoidosis: Japanese Society of Nuclear Cardiology recommendations. Ann Nucl Med.

[8] von Schulthess GK, Steinert HC, Hany TF (2006). Integrated PET/CT: current applications and future directions. Radiology.

[9] Vaidyanathan S, Patel CN, Scarsbrook AF (2015). FDG PET/CT in infection and inflammation—current and emerging clinical applications. Clin Radiol.

[10] Manabe O, Ohira H, Hirata K (2019). Use of ^18^F-FDG PET/CT texture analysis to diagnose cardiac sarcoidosis. Eur J Nucl Med Mol Imaging.

[11] Manabe O, Koyanagawa K, Hirata K (2020). Prognostic value of ^18^F-FDG PET using texture analysis in cardiac sarcoidosis. JACC Cardiovasc Imaging.

[12] Hu LH, Betancur J, Sharir T (2020). Machine learning predicts per-vessel early coronary revascularization after fast myocardial perfusion SPECT: results from multicentre REFINE SPECT registry. Eur Heart J Cardiovasc Imaging.

[13] Rios R, Miller RJH, Hu LH (2022). Determining a minimum set of variables for machine learning cardiovascular event prediction: results from REFINE SPECT registry. Cardiovasc Res.

[14] Otaki Y, Miller RJH, Slomka PJ (2022). The application of artificial intelligence in nuclear cardiology. Ann Nucl Med.

[15] Nakajo M, Ojima S, Kawakami H (2021). Value of Patlak Ki images from ^18^F-FDG-PET/CT for evaluation of the relationships between disease activity and clinical events in cardiac sarcoidosis. Sci Rep.

[16] Terasaki F, Azuma A, Anzai T (2019). JCS 2016 guideline on diagnosis and treatment of cardiac sarcoidosis—digest version. Circ J.

[17] Morooka M, Moroi M, Uno K (2014). Long fasting is effective in inhibiting physiological myocardial 18F-FDG uptake and for evaluating active lesions of cardiac sarcoidosis. EJNMMI Res.

[18] Muser D, Santangeli P, Castro SA (2018). Prognostic role of serial quantitative evaluation of ^18^F-fluorodeoxyglucose uptake by PET/CT in patients with cardiac sarcoidosis presenting with ventricular tachycardia. Eur J Nucl Med Mol Imaging.

[19] Nioche C, Orlhac F, Boughdad S (2018). LIFEx: a freeware for radiomic feature calculation in multimodality imaging to accelerate advances in the characterization of tumor heterogeneity. Cancer Res.

[20] Brown PJ, Zhong J, Frood R (2019). Prediction of outcome in anal squamous cell carcinoma using radiomic feature analysis of pre-treatment FDG PET-CT. Eur J Nucl Med Mol Imaging.

[21] Johnson WE, Li C, Rabinovic A (2007). Adjusting batch effects in microarray expression data using empirical Bayes methods. Biostatistics.

[22] Orlhac F, Boughdad S, Philippe C (2018). A postreconstruction harmonization method for multicenter radiomic studies in PET. J Nucl Med.

[23] Kandolin R, Lehtonen J, Airaksinen J (2015). Cardiac sarcoidosis: epidemiology, characteristics, and outcome over 25 years in a nationwide study. Circulation.

[24] Sinagra G, Anzini M, Pereira NL (2016). Myocarditis in clinical practice. Mayo Clin Proc.

[25] Tuominen H, Haarala A, Tikkakoski A, Kähönen M, Nikus K, Sipilä K (2021). FDG-PET in possible cardiac sarcoidosis: right ventricular uptake and high total cardiac metabolic activity predict cardiovascular events. J Nucl Cardiol.

[26] Kaneko K, Nagao M, Yamamoto A, Sakai A, Sakai S (2023). FDG uptake patterns in isolated and systemic cardiac sarcoidosis. J Nucl Cardiol.

[27] Choudhury P, Allen RT, Endres MG (2021). Machine learning for pattern discovery in management research. Strateg Manag J.

[28] El-Sappagh S, Saleh H, Sahal R (2021). Alzheimer’s disease progression detection model based on an early fusion of cost-effective multimodal data. Future Gener Comput Syst.

[29] Chawla NV, Bowyer KW, Hall LO (2002). SMOTE: synthetic minority over-sampling technique. J Artif Intell Res.

[30] Chicco D, Shiradkar R (2023). Ten quick tips for computational analysis of medical images. PLOS Comput Biol.

[31] Cook JA, Ranstam J (2016). Overfitting. Br J Surg.

[32] Krizmaric M, Verlic M, Stiglic G (2009). Intelligent analysis in predicting outcome of out-of-hospital cardiac arrest. Comput Methods Programs Biomed.

[33] Hyun SH, Ahn MS, Koh YW (2019). A machine-learning approach using PET-based radiomics to predict the histological subtypes of lung cancer. Clin Nucl Med.

[34] Mosavi A, Hosseini FS, Choubin B (2020). Susceptibility prediction of groundwater hardness using ensemble machine learning models. Water.

[35] Demsar J, Curk T, Erjavec A (2013). Orange: data mining toolbox in Python. J Mach Learn Res.

[36] DeLong ER, DeLong DM, Clarke-Pearson DL (1988). Comparing the areas under two or more correlated receiver operating characteristic curves: a nonparametric approach. Biometrics.

[37] Suzuki K, Yisong C (2018). Artificial intelligence in decision support systems for diagnosis in medical imaging.

[38] Xu R, Kido S, Suga K (2014). Texture analysis on ^18^F-FDG PET/CT images to differentiate malignant and benign bone and soft-tissue lesions. Ann Nucl Med.

[39] Cheng L, Zhang J, Wang Y (2017). Textural features of ^18^F-FDG PET after two cycles of neoadjuvant chemotherapy can predict pCR in patients with locally advanced breast cancer. Ann Nucl Med.

[40] Limkin EJ, Reuze S, Carre A (2019). The complexity of tumor shape, spiculatedness, correlates with tumor radiomic shape features. Sci Rep.

[41] Su Y, Choi CE (2021). Effects of particle shape on the cushioning mechanics of rock-filled gabions. Acta Geotech.

[42] Blankstein R, Osborne M, Naya M (2014). Cardiac positron emission tomography enhances prognostic assessments of patients with suspected cardiac sarcoidosis. J Am Cardiol.

[43] Nakajo M, Jinguji M, Tani A (2021). Application of a machine learning approach for the analysis of clinical and radiomic features of pretreatment [^18^F]-FDG PET/CT to predict prognosis of patients with endometrial cancer. Mol Imaging Biol.

[44] Li Y, Zhang Y, Fang Q (2021). Radiomics analysis of [^18^F]FDG PET/CT for microvascular invasion and prognosis prediction in very-early- and early-stage hepatocellular carcinoma. Eur J Nucl Med Mol Imaging.

