# Supplementary material for: Machine learning approach using 18F-FDG-PET-radiomic features and the visibility of right ventricle 18F-FDG uptake for predicting clinical events in patients with cardiac sarcoidosis
Source: Jpn J Radiol. 2024 Mar 16;42(7):744–52. doi: 10.1007/s11604-024-01546-y (PMC11217075; doi:10.1007/s11604-024-01546-y)
Supplement: Supplementary file 1 — Supplementary file1 (DOCX 116 KB) [file 11604_2024_1546_MOESM1_ESM.docx]

| **Supplemental Table 1.** List of the 49 quantitative positron emission tomography-based radiomic features | |
| --- | --- |
| Matrix | Index |
| Shape and first-order features | SUV_max_ |
|  | SUV_mean_ |
|  | SUV_peak_ |
|  | Cardiac metabolic volume |
|  | Cardiac metabolic activity |
|  | Surface area |
|  | Sphericity |
|  | Asphericity |
|  | Compacity |
|  | Kurtosis |
|  | Skewness |
| Gray level co-occurrence matrix | Inverse difference |
|  | Angular second moment |
|  | Contrast |
|  | Correlation |
|  | Entropy |
|  | Dissimilarity |
| Neighborhood gray-tone difference matrix | Coarseness |
|  | Contrast |
|  | Busyness |
|  | Complexity |
|  | Strength |
| Gray level run length matrix | Sort-runs emphasis |
|  | Long-runs emphasis |
|  | Low gray level run emphasis |
|  | High gray level run emphasis |
|  | Short-run low gray level emphasis |
|  | Short-run high gray level emphasis |
|  | Long-run low gray level emphasis |
|  | Long-run high gray level emphasis |
|  | Gray level non-uniformity |
|  | Run length non-uniformity |
|  | Run percentage |
| Gray level size zone matrix | Small-zone emphasis |
|  | Large-zone emphasis |
|  | Low gray level zone emphasis |
|  | High gray level zone emphasis |
|  | Small-zone low gray level emphasis |
|  | Small-zone high gray level emphasis |
|  | Large-zone low gray level emphasis |
|  | Large-zone high gray level emphasis |
|  | Gray level non-uniformity |
|  | Normalized gray level non-uniformity |
|  | Zone size non-uniformity |
|  | Normalized zone size non-uniformity |
|  | Zone percentage |
|  | Gray-level variance |
|  | Zone size variance |
|  | Zone size entropy |

| **Supplemental Table 2.** Ranking of 49 radiomic features and the visibility RV ^18^F-FDG uptake for predicting adverse clinical events in patients with cardiac sarcoidosis | | |
| --- | --- | --- |
| Rank | Features | Decrease in Gini impurity |
| 1 | Surface area | 0.218590335 |
| 2 | NGTDM Coarseness | 0.139642967 |
| 3 | GLRLM_ Run length non-uniformity | 0.139642967 |
| 4 | Sphericity | 0.115666359 |
| 5 | GLRLM_ Short-run low gray level emphasis | 0.109818406 |
| 6 | Visibility of RV ^18^F-FDG uptake | 0.107867291 |
| 7 | CMV | 0.106894429 |
| 8 | GLRLM_ Gray level non-uniformity | 0.091104955 |
| 9 | Asphericity | 0.083502616 |
| 10 | Compacity | 0.083502616 |
| 11 | GLSZM_ Zone size variance | 0.078824254 |
| 12 | GLSZM_ Large-zone high gray level emphasis | 0.078824254 |
| 13 | GLSZM_ Large-zone emphasis | 0.078824254 |
| 14 | GLCM_Correlation | 0.078824254 |
| 15 | GLRLM_ Low gray level run emphasis | 0.078824254 |
| 16 | Kurtosis | 0.076485072 |
| 17 | CMA | 0.064789166 |
| 18 | NGTDM_Strength | 0.064204371 |
| 19 | GLSZM_ Normalized gray level non-uniformity | 0.057186827 |
| 20 | GLRLM_ Long-runs emphasis | 0.050169283 |
| 21 | GLCM_ Inverse difference | 0.050169283 |
| 22 | GLSZM_ Gray-level variance | 0.04432133 |
| 23 | GLSZM_ Small-zone high gray level emphasis | 0.04432133 |
| 24 | GLSZM_ High gray level zone emphasis | 0.04432133 |
| 25 | SUVmax | 0.04432133 |
| 26 | NGTDM_Busyness | 0.041397353 |
| 27 | SUVpeak | 0.041397353 |
| 28 | GLSZM_Zone size entropy | 0.038473376 |
| 29 | GLSZM_ Gray level non-uniformity | 0.038473376 |
| 30 | GLCM_ Angular second moment | 0.038473376 |
| 31 | GLSZM_ Small-zone emphasis | 0.03671899 |
| 32 | GLCM_Dissimilarity | 0.03671899 |
| 33 | GLSZM_ Normalized zone size non-uniformity | 0.032625423 |
| 34 | NGTDM_Complexity | 0.032625423 |
| 35 | GLSZM_ Small-zone low gray level emphasis | 0.029701447 |
| 36 | GLSZM_ Low gray level zone emphasis | 0.029701447 |
| 37 | GLRLM_ Low gray level run emphasis | 0.029701447 |
| 38 | GLSZM_ Zone percentage | 0.027362265 |
| 39 | NGTDM_Contrast | 0.027362265 |
| 40 | Skewness | 0.027362265 |
| 41 | GLRLM_ Short-run high gray level emphasis | 0.024438289 |
| 42 | GLRLM_ High gray level run emphasis | 0.024438289 |
| 43 | SUVmean | 0.024438289 |
| 44 | GLRLM_ Run percentage | 0.022099107 |
| 45 | GLRLM_ Sort-runs emphasis | 0.022099107 |
| 46 | GLCM_Contrast | 0.020929517 |
| 47 | GLSZM_ Large-zone low gray level emphasis | 0.020344721 |
| 48 | GLCM_Entropy | 0.013911973 |
| 49 | GLSZM_Zone size non-uniformity | 0.012742382 |
| 50 | GLRLM_ Long-run high gray level emphasis | 0.006309634 |
| *RV*, right ventricle; *GLRLM*, gray level run length matrix; *CMA*, cardiac metabolic activity; *NGTDM*, neighborhood gray-tone difference matrix; *GLSZM*, gray level size zone matrix; *GLCM*, gray level co-occurrence matrix; *CMV*, cardiac metabolic volume; *SUV_mean_*, mean standardized uptake value; *SUV_peak_*, peak standardized uptake value; *SUV_max_*, maximum standardized uptake value | | |

| **Supplemental Table 3**. *p* values for comparing each ML model in each diagnostic index for predicting adverse clinical events in patients with cardiac sarcoidosis | | | | | | | | | | | | | | | | | | | | | |
| --- | --- | --- | --- | --- | --- | --- | --- | --- | --- | --- | --- | --- | --- | --- | --- | --- | --- | --- | --- | --- | --- |
|  | Decision tree | | | | | | RF | | | | | NN | | | | kNN | | | NB | | LR |
|  | RF | NN | kNN | NB | LR | SVM | NN | kNN | NB | LR | SVM | kNN | NB | LR | SVM | NB | LR | SVM | LR | SVM | SVM |
| Sensitivity | 1.00 | 1.00 | 1.00 | 1.00 | 1.00 | 1.00 | 1.00 | 1.00 | 1.00 | 1.00 | 1.00 | 1.00 | 1.00 | 1.00 | 1.00 | 1.00 | 1.00 | 1.00 | 1.00 | 1.00 | 1.00 |
| Specificity | 1.00 | 1.00 | 1.00 | 1.00 | 1.00 | 1.00 | 1.00 | 1.00 | 1.00 | 1.00 | 1.00 | 1.00 | 1.00 | 1.00 | 1.00 | 1.00 | 1.00 | 1.00 | 1.00 | 1.00 | 1.00 |
| PPV | 0.41 | 1.00 | 1.00 | 1.00 | 1.00 | 1.00 | 0.41 | 0.41 | 0.41 | 0.41 | 0.41 | 1.00 | 1.00 | 1.00 | 1.00 | 1.00 | 1.00 | 1.00 | 1.00 | 1.00 | 1.00 |
| NPV | 0.90 | 1.00 | 1.00 | 1.00 | 1.00 | 1.00 | 0.90 | 0.90 | 0.90 | 0.90 | 0.90 | 1.00 | 1.00 | 1.00 | 1.00 | 1.00 | 1.00 | 1.00 | 1.00 | 1.00 | 1.00 |
| Accuracy | 1.00 | 1.00 | 1.00 | 1.00 | 1.00 | 1.00 | 1.00 | 1.00 | 1.00 | 1.00 | 1.00 | 1.00 | 1.00 | 1.00 | 1.00 | 1.00 | 1.00 | 1.00 | 1.00 | 1.00 | 1.00 |
| AUC | 0.11 | 0.11 | 1.00 | 0.79 | 0.59 | 0.84 | 1.00 | 0.35 | 0.40 | 0.17 | 0.36 | 0.35 | 0.40 | 0.17 | 0.36 | 0.63 | 0.33 | 0.63 | 0.21 | 0.48 | 0.48 |
| *RF*, random forest; *NN*, neural network; *kNN*, k-nearest neighbors; *NB*, Naïve Bayes; *LR*, logistic regression; *SVM*, support vector machine, *PPV*, positive predictive value; *NPV*, negative predictive value | | | | | | | | | | | | | | | | | | | | | |


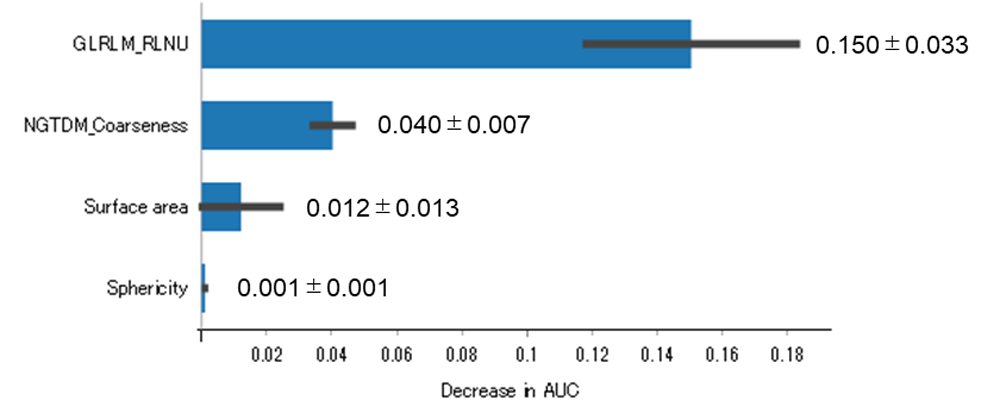


**Supplemental Figure 1.** Impact of individual factors scaled (by decrease in AUC) on the model output of the RF ML algorithm.

GLRLM_RLNU is the most important feature with the highest mean value (0.150), and this feature had a higher contribution in the modeling process.

**Supplemental material**

**Sphericity**

Sphericity is a measure to describe the sphere-like shape of the VOI [1].

Sphericity =$\frac{\sqrt[3]{36\pi V^{2}}}{A}$

V is defined as volume and A as surface area of the VOI. A is defined as the sum of length times width of every plane in the VOI.

**References**

1. van Helden EJ, Vacher YJL, van Wieringen WN, et al. Radiomics analysis of pre-treatment [^18^F]FDG PET/CT for patients with metastatic colorectal cancer undergoing palliative systemic treatment. Eur J Nucl med Mol Imaging. 2018;45:2307-17.
